# Acquired aplastic anaemia after SARS-CoV-2 infection in China: a case report

**DOI:** 10.3389/fped.2023.1277540

**Published:** 2023-11-07

**Authors:** Xiyan Wu, Yi Mo, Kailiang Wen, Rui Ming, Xinyu Yin, Liang Hu, Gang Liu, Ge Lin, Weina Li

**Affiliations:** ^1^ Center for Reproductive Medicine, Hunan Provincial Maternal and Child Health Care Hospital, Changsha, China; ^2^ Department of Laboratory Medicine, Hunan Children’s Hospital, Changsha, China; ^3^ Medical Laboratory, The Second Xiangya Hospital of Central South University, Changsha, China; ^4^ The Institute of Reproductive and Stem Cell Engineering, School of Basic Medical Science, Central South University, Changsha, China; ^5^ Department of Andrology, Reproductive and Genetic Hospital of CITIC-Xiangya, Changsha, China; ^6^ Clinical Research Center for Reproduction and Genetics in Hunan Province, Changsha, China; ^7^ Scientific Research Department, Hunan Guangxiu Hi-Tech Life Technology Co., Ltd., Changsha, China

**Keywords:** COVID-19, SARS-CoV-2, acquired aplastic anaemia, leukaemia, China

## Abstract

Since the coronavirus disease 2019 (COVID-19) pandemic began, several research groups in different countries have described cases of aplastic anaemia (AA) after COVID-19 or COVID-19 vaccination. Here, we present the case of a patient with new-onset AA in Changsha, China, that was presumably associated with preceding severe acute respiratory syndrome coronavirus 2 (SARS-CoV-2) infection. We conducted an epidemiological assessment of the incidence rate of blood system diseases from July 1, 2022, to May 31, 2023, in the haematology department of the Second Xiangya Hospital of Central South University and Hunan Children's Hospital. The detection rates of AA and leukaemia in the first two months after the epidemic outbreak were higher than those before and during the outbreak. However, only the difference in the detection rate of leukaemia was statistically significant.

## Introduction

Coronavirus disease (COVID-19) is a global infectious disease caused by severe acute respiratory syndrome coronavirus 2 (SARS-CoV-2). Since the COVID-19 pandemic began, several research groups have described cases of aplastic anaemia (AA) after COVID-19 or COVID-19 vaccination in Japan (1, 2), South Korea (3), Italy (4, 5), China Taiwan (6), the United Kingdom (7) and the United State (8–10). AA, a rare life-threatening disorder, is a syndrome of bonemarrow failure characterized by peripheral blood pancytopaenia and empty bone marrow (11). Acquired AA is a rare disease that affects approximately 2 people per million per year in North America and Europe, which is two to three times greater than the rate in Asia. Adolescents, young adults and elderly individuals are the age groups that are most affected, and the rates are roughly the same for men and women.We believe this is the first new case of acquired bone marrow failure potentially caused by SARS-CoV-2 infection reported in China. In addition, we conducted an epidemiological assessment of the incidence rate of blood system diseases from July 1, 2022, to May 31, 2023, in the haematology department of the Second Xiangya Hospital of Central South University and Hunan Children's Hospital.

## Patients and methods

A retrospective review of data from the Second Xiangya Hospital of Central South University and Hunan Children's Hospital, Changsha, Hunan, China, was performed. The number of patients with AA or leukaemia encounters was compared among half a year prior to the outbreak of COVID-19 (2022.7–2022.11), during the epidemic outbreak period (2022.12), the first two months after the outbreak of the epidemic (2023.1–2023.2) and later after the outbreak of the epidemic (2023.3–2023.5).Collection and retrospective analysis of anonymized data were approved by the institutional review board.

All statistical analyses were performed using IBM SPSS Statistics (v. 19.0; IBM Corp., Armonk, NY, USA).Categorical variables were summarized as frequency counts and percentages. Comparison of the detection rates of AA and leukaemia was performed using the chi-square test. All statistical tests were two-sided. *P*-values less than 0.05 were considered to indicate significance.

## Results

### Case presentation

A 14-year-old female was reported to have a previous history of good physical health, with menarche at age 11years and a menstrual cycle of 28–29 days and 4–5 days of ovulation. She was given two doses of the inactivated SARS-CoV-2 vaccine on August 14, 2021, and September 15, 2021, from Beijing Biotech and Chengdu Biotech, respectively. The complete blood count was normal in September 2022. There was no history of contact with toxins, nor was there a similar situation in the family. Amid the epidemic of the Omicron variant in December 2022 in Changsha, China, she tested positive for SARS-CoV-2 via polymerase chain reaction (PCR). She had a high fever of 39 degrees Celsius, lasting for 8–9 h, and was given oral acetaminophen (1 tablet at a time, 3 times per day) to reduce the fever. No routine blood test was performed at that time. On January 1, 2023, menstruation began and lasted for more than 10 days without a clean menstrual cycle. She fainted on January 11, 2023, and was admitted to the hospital. Blood tests showed pancytopaenia, and laboratory analysis revealed the following: a white blood cell (WBC) count of 0.3 × 10^9^/L, a red blood cell (RBC) count of 2.88 × 10^9^/L, an absolute neutrophil count (ANC) of 0.01 × 10^9^/L, an absolute lymphocyte count (ALC) of 0.29 × 10^9^/L, a haemoglobin (Hgb) level of 84 g/L, a reticulocyte count of 4.3 × 10^9^/L, and a platelet count of 46 × 10^9^/L. Her extensive workup, including for malignancy (including thoracic and abdominopelvic CT imaging), infection (HIV, viral hepatitis, Epstein‒Barr virus, and cytomegalovirus), and autoimmune aetiologies, was negative; vitamin B12 and folate levels were unremarkable. She was diagnosed with acquired AA according to laboratory criteria based on myelocytopaenia and total haemocytopaenia, excluding other causes of total haemocytopaenia and hereditary bone marrow failure, and after a first and second bone marrow biopsy, it was determined that her bone marrow cell counts were decreased by 10%. Extremely low bone marrow hyperplasia and adipose tissue hyperplasia were observed. Hematopoietic granulocytes and erythrocytes had low proliferation and were mainly composed of middle- and late-stage cells with a scattered distribution. No megakaryocytes were found on any slides. Individual lymphocytes and plasma cells were visible but scattered. Scattered tissue cells containing engulfed hemosiderin granules were clearly observed. No fibrosis was observed. Gomori staining results were MF-0 grade, and iron staining results were 2+. A comprehensive evaluation of inherited (including cytogenetic array, whole exome sequencing (WES), and copy number variation (CNV) sequencing) and acquired aetiologies of AA did not reveal the aetiology of this patient. During admission, this patient relied on oxygenated Hgb and platelet transfusion. Laboratory analysis on March 3, 2023, revealed the following: a WBC count of 0.4 × 10^9^/L, a RBC count of 2.08 × 10^9^/L, an ANC of 0.00 × 10^9^/L, an ALC of 0.3 × 10^9^/L, a Hgb level of 362.0 g/L, and a platelet count of 47 × 10^9^/L. On the same day, we detected SARS-CoV-2 nucleic acid positivity in this patient's blood cells and plasma. At the same time, six women who had never been infected with SARS-CoV-2 and six women who had recovered from COVID-19 in mid-December 2022 served as negative control groups, and their blood cells and plasma test results were negative. She received a bone marrow transplant from her father on April 1, 2023. At the time of reporting, this patient is in remission with a good haematologic response.

Epidemiological assessment of the incidence rate of blood system diseasesbefore and during the outbreak in Changsha, China.

To our knowledge, only 9 studies have reported 17 cases of blood system disease acquired after COVID-19. We provide a brief review of these cases in Table 1. Therefore, it has become particularly important for us to monitor adverse events after COVID-19. Notably, the omicron variant outbreak rapidly spread around China beginning in December 2022. According to the Chinese Center for Disease Control and Prevention, the number and positive rate of SARS-CoV-2 nucleic acid tests in all provinces showed a trend of first increasing and then decreasing; the number of positive results peaked on December 22, 2022 (6.94 million), and the positive rate peaked on December 25, 2022 (29.2%). We conducted an epidemiological assessment of the incidence rate of blood system diseases from July 1, 2022 to May 31, 2023 in the haematology department of the Second Xiangya Hospital of Central South University and Hunan Children's Hospital (Table 2).The detection rates of AA and leukaemia in the first two months after the outbreak of the epidemic were higher than those before and during the outbreak. However, only the difference in the detection rate of leukaemia was statistically significant.

**Table 1:** Reported cases of newly diagnosed blood system diseases after COVID-19 infection.

Age (y)	Sex	Country	Symptoms	Time of disease occurrence after infection with COVID-19	Therapeutic treatment	Treatment outcome	Diagnostic results	Possible mechanism	Reference
78	Female	Italy	Asthenia and fatigue	NA	NA	Improvement	AA and PNH	NA	Iannuzzi et al. (5)
NA (3 persons)	NA	UK	Fatigue, generalmalaise, fever, dry cough, shortness of breath, loss ofsmell, and diarrhea	A few weeks later	IST or hematopoietic stem cell transplantation	Good response	Severe or very severe AA	Potential myelosuppressive effect of SARS-CoV-2	Avenoso et al. (7)
12	Female	USA	Pallor, fatigue, andchest tightness	NA	IST with antithymoglobulin (ATG) and cyclosporine	Good response	SevereAA	Novel coronavirus may contribute to the typicalimmune-mediated pathogenesis of SAA	Chakravarthy et al. (8)
18	Male	USA	Fatigue, headache, and fever	NA	IST with ATG and cyclosporine	Good response.	SevereAA	Novel coronavirus may contribute to the typicalimmune-mediated pathogenesis of SAA	Chakravarthy et al. (8)
5 months	Female	USA	Fever (39.6°C) and intermittent tachycardia	NA	Methylprednisolone treatment	Good response	MIS-C	NA	Mariani et al. (9)
22	Female	USA	Fatigue and ecchymoses	10 days	Stem cell transplant	Good response	AA	SARS-CoV-2 may mediate an immunologic response	Lee et al. (10)
69	Female	Asia	Fatigue andpancytopenia	NA	Cyclosporine, h-ATG, and eltrombopag	Improvement	AA	SARS-CoV-2 may mediate an immunologic response	Lee et al. (10)
76	Male	USA	Profound anemia,chest pain	NA	Tacrolimus	Good response	PRCA	SARS-CoV-2 may mediate an immunologic response	Lee et al. (10)
NA	NA	NA	Fever, chills, and sorethroat	NA	Cyclosporine and eltrombopag, h-ATG.	Good response	Severe AA and pancytopenia withsubclinical paroxysmal nocturnal hemoglobinuria (PNH) clones and COVID-19 infection	SARS-CoV-2 may mediate an immunologic response	Lee et al. (10)
69	Female	NA	Severe pancytopenia	5 months	Cyclosporine, h-ATG, and eltrombopag	Improvement	Platelet transfusion–dependent severe AA, PNH	SARS-CoV-2 may mediate an immunologic response	Lee et al. (10)
28	Female	NA	Pancytopenia	3 months	Cyclosporine, h-ATG, and eltrombopag;achieved transfusion independence	Improvement	Severe AA	SARS-CoV-2 may mediate an immunologic response	Lee et al. (10)
75	Male	NA	Fever and tachycardia	NA	NA	Improvement	MBCL	NA	Lanza Let al. (12)
55	Male	NA	Shortness of breath, dry cough, and fever	NA	Dexamethasone, remdesivir and tocilizumab	Improvement	CLL	NA	Saluja et al. (13)
49	Male	NA	Shortness of breath, fever, and body aches	NA	Oseltamivir,azithromycin, hydroxychloroquine,ceftriaxone and oral amoxicillin/clavulanate	Good response	Early-stage CLL	NA	Ali et al. (14)
83	Male	NA	Fever, nonproductive cough, dyspnea, and rectal bleeding	NA	Paracetamol, amoxicillin, and clavulanic acid	Improvement	CLL	NA	Largeaud et al. (15)

AA, Aplastic anemia; MIS-C, multisystem inflammatory syndrome in children; PRCA, pure red cell aplasia; HSCT, hematopoietic stem cell transplantation; PNH, paroxysmal nocturnal hemoglobinuria; SARS-CoV-2, severe acute respiratory syndrome coronavirus 2; IST, immunosuppressive therapy; ATG, antithymoglobulin; h-ATG, equine antithymocyte globulin; MBCL, monoclonal B-cell lymphocytosis; CLL, chronic lymphocytic leukemia.

**Table 2:** Assessment of the incidence rates of leukemia and aplastic anemia in two hospitals in Changsha.

Names of hospitals	Detection rate (%, *N*/*n*)	Stage 1 2022.7.1-2022.11.30	Stage 2 2022.12.1–2022.12.31	Stage 3 2023.1.1–2023.2.28	Stage 4 2023.3.1–2023.5.31	*P*-value
Hunan Children's Hospital	Leukemia	3.31 (53/1,601)	2.39 (5/209)	4.66 (27/580)	3.74 (37/989)	0.366
	Aplastic anemia	1.12 (18/1,601)	0.96 (2/209)	1.55 (9/580)	2.02 (20/989)	0.278
The Second Xiangya Hospital of Central South University	Leukemia	7.20 (174/2,417)	3.70 (9/243)	8.52 (66/775)	6.58 (99/1,505)	0.063
	Aplastic anemia	0.91 (22/2,417)	0.82 (2/243)	1.03 (8/775)	0.73 (11/1,505)	0.891
Total	Leukemia	5.65 (227/4,018)	3.10 (14/452)^a^	6.86 (93/1,355)^b^	5.45 (136/2,494)^b^	0.024
	Aplastic anemia	1.00 (40/4,018)	0.88 (4/452)	1.25 (17/1,355)	1.24(31/2,494)	0.717

*N*, diagnosed with leukemia or aplastic anemia; *n*, number of bone marrow smears.

^a^ *p *< 0.05 for comparison with stage 1.

^b^ *p *< 0.05 for comparison with stage 2.

## Discussion

According to some recent reports, SARS-CoV-2 infection precedes the occurrence of some autoimmune diseases and haematological diseases, including paediatric inflammatory multisystemic syndrome (PIMS), systemic lupus erythematosus (SLE), immune thrombocytopenia, chronic lymphocytic leukaemia and acquired haemophilia. Most of these reports have linked COVID-19 to the development of these diseases based on clinical observations of temporal associations. The possible main mechanisms linking COVID-19 and AA are as follows: 1. excessive production of inflammatory cytokines and the occurrence of cytokine storm in COVID-19 (16); 2. cytokine storm and the potential cytotoxicity of the virus (17, 18); 3. abnormal hematopoiesis caused by SARS-CoV-2 through the infection of bone marrow erythroid cells (19, 20); 4. an aberrant immune response triggered by the virus leading to depletion of the stem cell compartment and inducing bone marrow failure (7, 10); 5. a potential myelosuppressive effect of this virus (21, 22, 23); 6. manipulation of the hematopoietic stem cell replication process by impairing the expression of several vital proteins as well as disrupting the intracellular biochemical cascade (24, 25); 7. abnormal erythropoiesis caused by CD147on hematopoietic cellsacting as a receptor for virus entry (26); and 8. direct infiltration of the virus into the bone marrow (27).

To our knowledge, this is the first new case of acquired bone marrow failure reported in China. This patient had a very short time (15 days) between PCR positivity and total blood cell decline. This presentation is consistent with previous infectious disease data showing that SARS-CoV-2 infection leads to immune-mediated bone marrow failure, and the infection precedes pancytopenia by weeks to months. Considering the temporal correlation, it appears possible that COVID-19 had a direct role in the pathogenesis of AA.

Immunodeficient or immunocompromised persons are at risk of a prolonged viral phase, with a contagious period of shedding infectious viral particles that can even last for months compared to the typical 5-10 days reported for the general population. This patient remains persistently positive on PCR testing of blood cells and plasma even after 3 months of infection. This supports the theory that SARS-CoV-2 may be causally associated with AA.

However, due to the lack of cytokine studies or viral PCR analysis of bone marrow aspirates in our study, it has not been clarified whether the virus has a direct cytotoxic effect on haematopoietic stem cells or acts through a cytokine storm or aberrant immune dysregulation following the infection, which caused this patient to develop secondary AA. Further evaluations in large cohorts are warranted to elucidate the associations between blood system diseases and COVID-19.

## Data Availability

The raw data supporting the conclusions of this article will be made available by the authors, without undue reservation.
